# “Design and implementation challenges of massive open online course on research methods for Indian medical postgraduates and teachers –descriptive analysis of inaugural cycle”

**DOI:** 10.1186/s12909-022-03423-6

**Published:** 2022-05-13

**Authors:** Manickam Ponnaiah, Tarun Bhatnagar, Parasuraman Ganeshkumar, Ditipriya Bhar, Rajalakshmi Elumalai, Mathavaswami Vijayageetha, Rizwan Suliankatchi Abdulkader, Sirshendu Chaudhuri, Upasana Sharma, Manoj Vasant Murhekar

**Affiliations:** ICMR-National Institute of Epidemiology, R127, TNHB, Ayappakkam, Chennai, Tamil Nadu 600077 India

**Keywords:** Descriptive analysis, E-learning, International medical education, Medical education research, MOOCs, Teaching learning methods

## Abstract

**Background:**

In view of the growing popularity, reach and access for Massive Open Online Courses (MOOCs), India’s apex body for medical education, the National Medical Commission (NMC) mandated uniform foundational course on research methods for the medical post graduates (PGs) and faculty members of the medical institutions under NMC as MOOC. This course is a pioneering effort in the field of India’s PG medical education. NMC entrusted Indian Council of Medical Research (ICMR)-National Institute of Epidemiology (NIE) to design and offer the MOOC, named as Basic Course in Biomedical Research (BCBR). We describe the experience of designing and that of implementation challenges in the inaugural cycle of the course.

**Methods:**

The course objective was to inculcate the fundamental concepts in research methods covering epidemiology and biostatistics in the form of video lectures, resource materials, discussion forum, assignments, feedback and a final proctored examination. The course was delivered over 16 weeks through MOOCs platform under the Indian Ministry of Education. We reviewed records, documents and faculty notes and described the course conceptualization, development, design and implementation process. We abstracted information from course portal on enrolment profile of the participants, self-reported course feedback (structured and open-ended on format, lectures and quality of contents), examination registration form, scores obtained in the assignments/examination and that of the participant queries. We described quantitative data using descriptive statistics. We presented the thematic analysis of qualitative data from open-ended questions in the feedback system and that of email interactions.

**Results:**

The inaugural cycle (September-December 2019) was taken by 24,385 participants. Majority, 15,879 (65%) were from medical background. 13,242 (54%) were medical postgraduates and 2637 (11%) were medical teachers. Among the enrolled, 14,720 (60%) cleared the assignments. A total of 11,392 (47%), 8,205 (62%) medical PGs and 896 (34%) faculty members successfully completed the course. Feedback from 1305 (5%) participants had mean score of 4.5/5 (±0.7) for quality of teaching. We faced challenges in customizing the course for medical participants, unawareness among target group, digital illiteracy and the ongoing pandemic.

**Conclusions:**

During the inaugural cycle of the online Basic Course in Biomedical Research course, nearly half of the enrolled participants successfully completed and received the certificate. India’s MOOC for enhancing research capabilities of future medical researchers encountered successes and challenges. Lessons learnt from the inaugural cycle will guide future directions and to address larger issues in terms of sustainability and replication by stakeholders in medical education in India or elsewhere.

**Supplementary Information:**

The online version contains supplementary material available at 10.1186/s12909-022-03423-6.

## Background

Health research is a prerequisite for pioneering advancements in medicine [1]. In developed countries, there is extensive investment to identify and investigate health research priorities. In India, although health research has surged in the last decade, it is clustered within few institutions with excessive focus in specific geographical settings. The dearth of forums to interact and share knowledge, inaccessibility of existing global resources and information asymmetry all pose a threat to the ecosystem of health research [2]. A lack of uniform training programme in research methods is one of the key factors posing a threat to both quantity and quality of research conducted in India [3]. By using online teaching tools such as MOOCs , we can impart knowledge, and skill in research methods.

The way education has been taught and practiced has changed since the advent of digital technology [4]. Recently, Massive Online Open Courses (MOOCs) have seen an increase in their development and implementation across a wide array of sectors of higher education, including health professions education [5, 6]. MOOCs gained popularity due to its flexibility and accessibility to provide content anywhere and anytime with a meaningful learning experience [7]. Studies have shown that MOOCs are highly efficient and effective for learning [8–10]. There is enough evidence that e-learning can achieve the same level of effectiveness as traditional didacticism and promote self-directed learning [11].

According to a review of current MOOCs, a major proportion are now provided by institutions in developed countries [12]. Globally, while major emphasis on the student experience is understandable, there appears to be little research on the provider experiences and challenges [13]. Several reviews of MOOC literature have been done in order to identify emerging trends and directions for future research into the characteristics that can help MOOCs achieve long-term success [10, 14, 15]. There have been very few studies on the implementation and uptake of MOOCs, as well as the implications of the adoption of MOOCs in countries like India.

The National Medical Commission (NMC) (erstwhile Medical Council of India), India’s apex body for regulating medical education, recognizes and recommends medical research as an integral part of the post-graduate (PG) medical education. The key objectives of the recommendation are to develop competency in basic concepts of research methodology and epidemiology, critical appraisal of published research and function as an effective leader of a health team engaged in health care, research and training. Despite the large number of theses being conducted by these postgraduates, the quality of their work remains questionable. The capacity of the medical teachers who guide PG theses is also inadequate. Strengthening the teaching of research methods in PG medical curriculum could improve the current status. This huge research machinery offers an opportunity for good research that can influence practice or contribute to health programmes [16, 17].

Keeping the above in context, the NMC decided to introduce a mandatory, uniform, online, foundational course on research methods for the medical PGs and teachers in the country. The NMC identified the National Institute of Epidemiology (ICMR-NIE), an Institute under the Indian Council of Medical Research (ICMR) to develop and deliver an online course on fundamental concepts in research methodology for medical postgraduates and faculty members across the nation [18]. The selection of ICMR-NIE was based on the institute’s prior experience in developing and delivering massive open online courses (MOOCs) on health research through the MOOCs programme of the Indian Ministry of Education, called SWAYAM [‘Study Webs of Active-Learning for Young Aspiring Minds’]. Building on the experience of ICMR-NIE, the present course called Basic Course in Biomedical Research (BCBR) was designed and delivered through the SWAYAM. In the context of limited reports on the experiences and challenges associated with the implementation of MOOCs for improving biomedical research capacity in medical schools from the Indian sub-continent and that of similar countries, we conducted a retrospective study and described the experience of designing and that of implementation challenges in the inaugural cycle of the BCBR.

## Methods

The course objective was to inculcate the fundamental concepts in research methods covering epidemiology and biostatistics in the form of video lectures, resource materials, discussion forum, assignments, feedback and a final proctored examination. The course was delivered over 16 weeks through MOOCs platform under the Indian Ministry of Education. The inaugural cycle (September-December 2019) was taken by 24,385.

### Study approach

We did a retrospective study to analyse the inaugural cycle of the course based on review of course records and that of the data generated during the implementation process.

### Data collection

In order to describe the course conceptualization, development, design and implementation process, we reviewed records, documents and faculty notes.

We accessed the course portal for abstracting data on enrollment profile of the participants, assignment completion with scores, examination registration form and scores in the assignments and performance in the proctored examination. The course collected feedback of the participants on course contents, structure, faculty, lecture presentation and quality of contents through a 5-point Likert scale ranging from “strongly agree” to “strongly disagree”. We accessed the feedback data for every module and that of overall experience at the end of the course. We reviewed emails sent by course participants regarding queries related to administrative and technical aspects.

Additionally, as part of the course feedback, for each of the module, we collected response to three open-ended questions on what the participant liked, inputs and suggestions to improve and key learning points. We abstracted qualitative data from these open-ended questions, from discussion forum and that of email queries and interactions of the participants.

### Data analysis

Based on the review of the documents and notes, we identified the core components of the course, in terms of structure, content and the implementation process of the course. We enlisted the challenges, barriers and facilitators faced in the inaugural cycle of the course.

The quantitative data was used to describe the characteristics of the participants by age, educational background and profession. We plotted number of Indian participants by their Indian State. We calculated percentage assignment submission, mean and standard deviation (SD) of scores of the assignments by lecture and pass percentage. Participants who obtained at least 50% in all the assignments put together were eligible to register for the final proctored examination. We calculated descriptive statistics for participants’ feedback in various domains. We calculated the mean score and standard deviation for the Likert type questions.

We did analyze manually the contents of the qualitative data from the feedback system and that of email interactions. Codes were identified which were then categorized into emergent themes by two authors (ER and SC) independently. Any difference between them was resolved by RA through independent review. All of them were trained in qualitative research. In addition, a word cloud was created for conceptual analysis for the most commonly occurring word in the verbatim texts.

## Results

### Conceptualization, development, design and implementation process of the course

#### Learning objectives of the course

The objective of the course was to explain fundamental concepts in epidemiology and biostatistics. Inaugural cycle of BCBR course (September-December 2019, cycle 1) was conducted for 16 weeks by the ICMR-NIE and was delivered through one of the national coordinators of the SWAYAM, namely NPTEL (National Programme on Technology Enhanced Learning) located at the Indian Institute of Technology-Madras, Chennai.

#### Organizational structure and functions

At the national level, the course coordination committee established by the NMC was monitoring the progress of the course. At the Institute level, the course is coordinated by a core team under the supervision of the Director of ICMR-NIE. The Indian Council of Medical Research (ICMR) funded the initiative. The course is supported by a group of teaching and non-teaching staff of ICMR. The team has been divided into administrative and academic wings. The administrative wing is responsible for handling the issues related to the enrolment and registration of the course participants with a dedicated helpline number and an email account. The academic wing facilitates subject matter query resolution and updating the academic content

#### Course development team

The NMC formed an expert committee to meet the vision of the “Postgraduate Medical Education (Amendment) Regulations, 2019” to instill a spirit of scientific inquiry, orient the principles of research methodology and epidemiology by the medical postgraduates [19]. ICMR-NIE's multi-disciplinary team of epidemiologists, biostatisticians and public health specialists developed the curriculum contents of BCBR (Additional table 1) according to the competency matrix given by NMC for research methods. This was designed as a self-paced MOOC, as per the SWAYAM guidelines [20]. As per the official notification of NMC dated 13 December 2019, medical PGs had to mandatorily complete the BCBR.

**Table 1:** Participant performance in lecture-specific assignments in a massive open online course on biomedical research in India, 2019-2020 (*N*=15,879)

**Lectures**	**Medical PGs (*N*=13,242)**			**Medical faculty (*N*=2637)**		
	**% Submission**	**% Pass**	**Score [Mean (SD)]**	**% Submission**	**% Pass**	**Score [Mean (SD)]**
Introduction	82.1	99.6	89.4 (10.4)	46.3	99.5	88.9 (11.1)
Research question	81.5	99.1	88.2 (11.1)	43.9	98.4	86.9 (13.7)
Literature review	81.3	99.7	93.4 (9.6)	43.1	99.6	91.1 (11.8)
Designing study	81.2	99.3	84.5 (12.8)	42.2	98.4	84.4 (14.5)
Disease frequency	81.2	98.9	81.0 (13.1)	41.9	97.1	79.5 (15.2)
Descriptive study	81.0	99.6	94.1 (9.8)	41.5	98.8	91.4 (12.8)
Experimental study	80.8	99.7	90.7 (9.7)	41.2	99.5	89.6 (10.8)
Validity	80.8	99.5	92.6 (10.4)	40.8	98.8	91.7 (12.7)
Qualitative research	80.6	98.9	92.3 (11.4)	40.4	97.5	88.7 (15.5)
Measurement of variables	80.7	99.7	94.8 (8.8)	40.5	99.1	91.2 (12.4)
Sampling	80.6	99.0	94.9 (11.0)	40.4	98.8	93.1 (12.2)
Sample size & power	80.4	98.7	86.2 (13.8)	40.2	97.9	84.1 (15.5)
Selection of study population	80.5	99.8	94.0 (7.9)	40.1	99.6	94.4 (8.8)
Study plan	80.5	99.8	94.9 (8.0)	39.8	99.4	94.4 (9.7)
Data collection tool	80.4	99.2	87.7 (11.1)	39.8	97.5	85.4 (15.0)
Data collection	80.3	99.9	96.5 (6.2)	39.8	99.4	96.8 (8.0)
Data management	80.3	99.8	92.2 (8.3)	39.8	99.3	90.7 (10.0)
Data analysis	80.2	99.7	94.3 (8.9)	39.7	98.8	92.6 (12.4)
Ethics	80.3	99.4	84.0 (10.0)	39.9	99.1	83.8 (11.5)
Clinical trials	80.3	99.3	88.2 (11.2)	39.8	99.4	87.5 (11.9)
Concept paper	80.2	99.8	91.3 (9.5)	39.7	99.3	92.4 (9.7)
Protocol writing	80.3	99.1	93.8 (11.4)	39.7	99.1	91.4 (12.7)
Publication ethics	80.0	99.0	92.7 (11.6)	39.3	99.6	95.5 (8.9)

#### Course contents

The recommended format follows the four-quadrant instructional design which includes: e-tutorial (course videos), e-contents (reading materials, lecture handouts, text transcripts), the discussion forum and assessment (assignments and proctored examination). Accordingly, the course included 23 lectures with an average duration of 20 minutes for each lecture and delivered across 16 weeks. The course covered the topics - conceptualizing a research study, epidemiological considerations and bio-statistical considerations in designing a research study, planning and conducting a research study, writing a research protocol and publication ethics. A total of 23 lectures were covered under these topics by experts in the field of biostatistics, epidemiology, public health, and research methods from ICMR-NIE.

#### Course implementation

Medical colleges were officially notified regarding the site and enrolment process by the NMC well in advance. The participants were notified through their medical colleges to enroll until 1^st^ December 2019 on the course website or on the SWAYAM mobile application. The enrollment was free of cost. On the course page, the administrative wing regularly posted course-related announcements. Enrolled participants received email and text messages reminders on due dates. They were also able to make inquiries through the helpline.

Upon enrolment, course participants were able to access the course content including the lecture videos, handouts of the slides, transcripts and the reference links. Additionally, the course page provided assignments after each lecture, announcements and frequently asked questions regarding the course process. For academic clarifications, a separate section namely ‘discussion forum’ was created where the participants could post lecture related queries to the teaching assistants (TA). The TAs resolved the queries within two working days.

#### Assessment and certification

The BCBR course offered both formative as well as a summative assessment to determine eligibility for certification. The assessment strategy adopted was in alignment with the learning objectives of the BCBR. For the formative assessment, the participants had to complete the online auto-graded assignments after every lecture. Each assignment consisted of ten multiple-choice questions. For each lecture, the academic team prepared a set of questions after multiple rounds of internal revisions. The course participants could submit the assignments any number of times before the deadline and the one submitted last was considered for scoring. A minimum of 50% score in the assignment was required to be eligible for final examination registration. Those scoring <50% were prompted to redo the assignments in the next BCBR cycle with the same login credentials.

For summative assessment, an in-person proctored computer based examination was to be conducted at 138 designated centers across the country. Eligible participants were directed to register for the proctored examination by paying a nominal fee using the registration link available on the course page. We initiated the registration process three months in advance of the scheduled examination dates. Six sets of question papers each consisting of a hundred multiple choice questions were prepared by a team of experts from ICMR-NIE. We had scheduled the exams for three days in March and April 2020 with two slots per day. However, the exams could not be conducted due to the ongoing pandemic.

A course participant was considered ‘pass’ and ‘eligible’ for a verifiable e-certificate only if s/he scored at least 50% on an average in the assignment score and final examination score separately. The final marks were communicated to the participants within four weeks from the date of completion of the proctored examination. In the event of scoring less than 50 percent in the proctored examination, the participants had to register again in the next BCBR cycle for the proctored examination with the same login credentials without re-submitting the assignments.

#### Quality assurance

Quality assurance mechanism was built into the design and conduct of the course. Through the ‘participant feedback mechanism’ participants were able to give their feedback after the completion of each lecture via a link provided below each video. Further inputs and suggestions from participants to improve the quality of course and key learning points from the sessions were documented. In the discussion forum, solutions to the academic queries were formulated by a team of experts including teaching assistants and course consultants. In addition to this, the finalization of the proctored examination was undertaken with strict adherence to the rules laid down by the NPTEL. An extensive technical review of the questions was undertaken by the teams from ICMR-NIE and NPTEL for the finalization of the six sets of encrypted question papers. Moreover, periodic interim meetings were organized between the course coordination committee, course faculty and consultants discussing the progress and challenges faced and the solutions.

### Description of the course participants

#### Enrolment

**Fig. 1:**
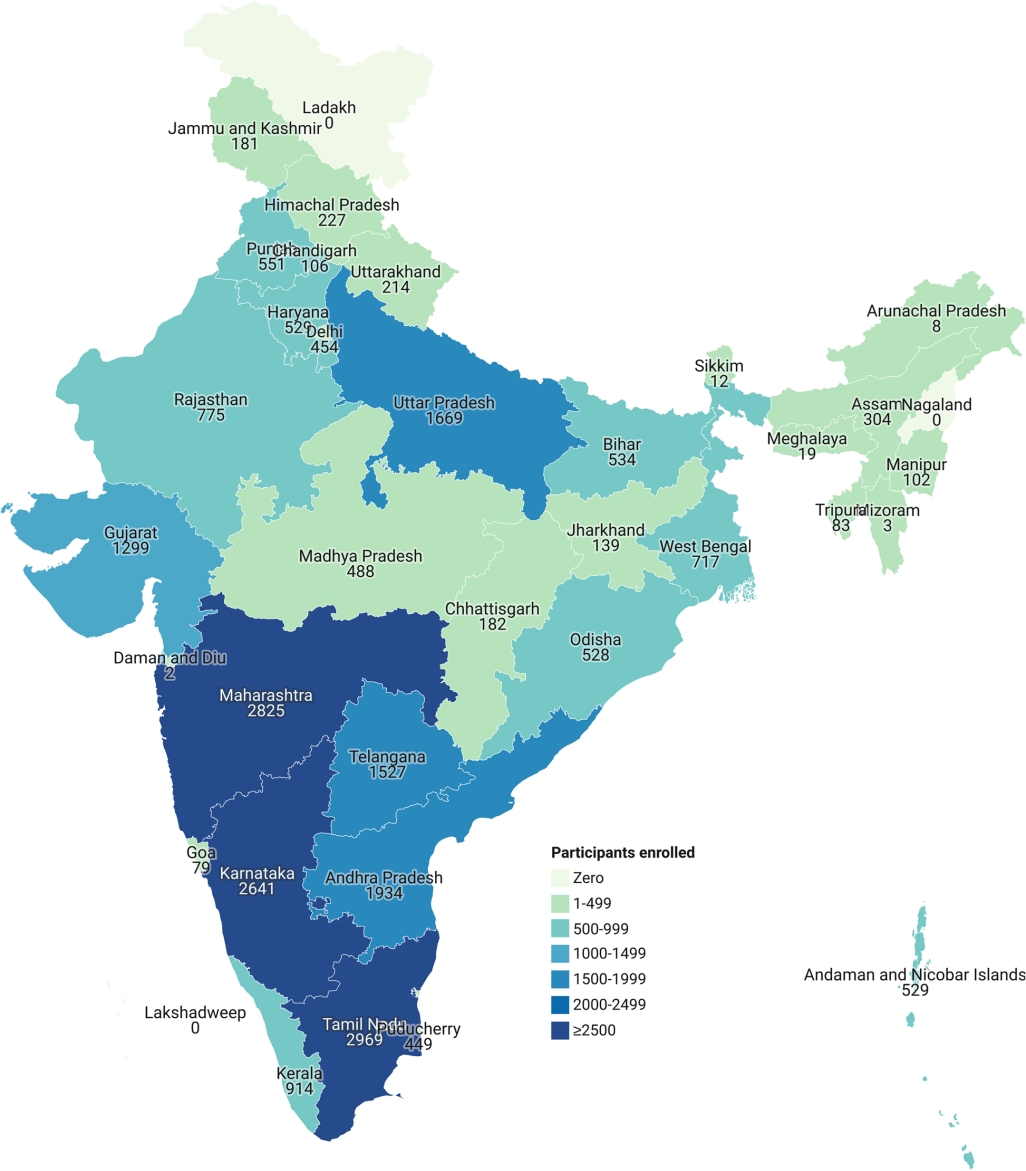
Geographical distribution of participants in a massive open online course on biomedical research in India, 2019-2020

A total of 24,385 participants from 38 countries enrolled during cycle 1. Majority (*n*=15906, 65%) were aged 21-30 years. Out of the total enrolled participants, 24,182 (99.2%) were from India. (Additional Table 2) Within India, majority of the participants were from Tamil Nadu and Maharashtra (12% each) states, followed by Karnataka (11%), and Andhra Pradesh (8%) (Fig. 1).

**Table 2:** Verbatim examples of qualitative feedback received from the participants in a massive open online course on biomedical research in India, 2019-2020

**What did you like about the lecture?** “*Relevant”* *“Basic”* *“Precise”* *“Topic is clearly understandable”* *“Explanation was apt”* *“Very much informative session”* *“Concise and relevant”* *“Presentation was simple and precise”* *“Good diagrams”*
**What could be improved?** *“More explanation is required for Relative risk and Odds ratio”* *“Data management software, Illustrate data entry in excel”* “*Working out an example for calculating risk and ratio*” “*more explanation for formulas”* *“Including image/picture of normal distribution curve for explaining hands on exercise confidence interval”*
**What could be added to the lecture?** *“Graphics more visuals and pictures”* *“Screenshots of good protocols”* *“Give a screenshot of a well prepared concept paper”* “*More clinical examples”* *“Animation”* *“More illustrative examples” “graphics”*

Among the participants, 13,242 (54%) were PGs pursuing MS or MD and 2,637 (11%) were faculty members. Among the medical PGs, 9,213 (69.6%) were pursuing MD and 4029 (30.4%) were pursuing MS. The remaining 8,506 (35%) participants were from outside the target group and included participants who were doing medical diploma, Master of Public Health (MPH) or employed elsewhere. (Additional Table 2)

Among the total enrolled, 14,720 (60%) had passed the assignments and qualified to register for the proctored exams. Of the medical PGs enrolled, 10,726 (81%) and among the faculty enrolled, 1055 (40%) passed the assignments and were eligible to register for the proctored examination. Among the total enrolled, over all 11,392 (47%), medical PGs 8205 (62%) and faculty 896 (34%) registered for the proctored examination. Due to the COVID-19 pandemic, in-person proctored exams could not be conducted. Therefore, the NMC decided to issue pass certificates to all those who had qualified from the assignments and were registered for the proctored examination.

In the analysis by lectures, among the faculty members, the frequency of completion gradually declined from lecture 1 to lecture 23. 38% of the faculty members completed all the assignments. Among those who submitted the assignments, the pass percentage varied from 97% (for lecture 5: Descriptive study designs) to 99.6% (for lecture 3: Literature review). Among PGs, the assignment completion rate varied from 77% to 82%, gradually declined from lecture 1 to lecture 23, and 77% completed all the assignments. Among those PGs who submitted, the pass percentage varied from 99.9% (lecture 16: Principles of data collection) to 99% (lecture 12: Sample size). When taking the mean score of the lectures into account, the five lowest-scoring lectures for both faculty members and PGs were lectures involving developing tools or bio statistical considerations. (Table 1)

A total of 1,845 queries were posted by the participants in the discussion forum which included both academic and administration related queries. There were approximately 348 (19%) academic queries and 1497 (81%) administrative queries which included 1,151 (62%) enrollment and technical, 112 assignment and eligibility related and 234 examination related queries.

### Feedback from participants

A total of 1,305 (5%) participants responded with feedback. The mean score (SD) of overall quality of teaching sessions was 4.5 (0.7). (Additional table 3) Majority of the course related technical queries were regarding accessing the course materials and registration in the examination. We implemented a number of solutions for these queries (Additional table 4). Most of the participants expressed their satisfaction about the content, clarity, explanation, presentation and the way the speakers delivered the lecture. A few suggested adding further explanation in certain topics and hands on training in biostatistics, specifically, sampling methods, sample size calculation, data management software and calculation of measures of association. For most of the lectures the participants suggested addition of more examples, especially with screenshots, graphics, animation and clinical scenarios. (Table 2, Additional Figure 1)

### Challenges faced

#### Implementation challenges

As with all major rollouts, the BCBR programme faced several challenges. These challenges can be organized into administrative, technical and participant related challenges. As described, the BCBR course depends on a smooth coordination between three major organizations. This resulted in a few delays in communications between them. In our course, decision making had to go through layers of communications at various levels. Evidence showed that inter-institutional collaborative efforts often impede the course development [21]. Despite these delays, the course was launched on the promised time and successfully started recruiting participants. Another administrative challenge was a lower than expected enrolment of first year postgraduates who were the intended participants for the course. It was realized later that the many PGs were unaware about the course. This was followed by widespread dissemination activities, where information was sent through proper channel to the college deans for dissemination within the college. Despite such activities, the enrolment was poor. Previous research suggested that recruitment of participants often reported as low when a course is initially launched [21]. This was alike to our experience in the course enrollment. There were some delays in responding to the participants. This delay took place because of two reasons. First, participants sometimes asked questions beyond the scope of the lectures. However, we earnestly addressed them with few exceptions. Second, most of the faculty members of BCBR are also researchers in the ICMR-NIE. Hence, there were delays in resolving the participant queries. Overall, most of the queries were answered within 48 hours. When it came to the stage of assignment completion and examination registration, there were a lot of dropouts.

The SWAYAM-NPTEL have been offering several science and technology courses. Offering this MOOC in customized way to a medical group under another authority was a challenge. Few participants faced difficulty in enrollment, accessing and submitting assignments. In other words, digital non-literacy was a major course hindrance factor. A few participants who decided to pay the examination registration fees on the last day of the registration faced difficulties in completing the payment.

#### Pandemic related challenges

Since most of our participants were doctors and were actively engaged in the Coronavirus disease 2019 (COVID-19) pandemic duties they could not complete the assignments/course. Some of the doctors were reassigned for COVID-19 duty and could not continue course. Requests for cancelling and changing of examination centres were placed by several participants. The examination which was scheduled in March/April 2020 was postponed and then finally cancelled.

#### Participant side challenges

Apart from the digital literacy challenges faced by the participants, there were issues related to internet connectivity in some areas of the country. Participants experienced problems in accessing the course contents and submitting the assignments. In some cases, the assignment submission did not register with the android app. For developing country like India technological infrastructure could be a major factor for successful retention of MOOCs participants [22].

To resolve the issue the BCBR team posted several announcements on the course page, mailed and texted the course participants individually. However, some of these technical challenges were beyond the scope of the ICMR-NIE to resolve and the NPTEL team had to be intimated regarding these issues. Therefore, maintaining the chain of communication between several stakeholders delayed solving of some issues.

## Discussion

In order to improve the rigor of teaching health research among medical postgraduates and the faculty members, India’s apex medical education regulatory body entrusted launching of MOOC on research methods through ICMR-National Institute of Epidemiology. Towards achieving the same, with full support from the ICMR, we designed and implemented a MOOC on research methods. We share our experience from the inaugural cycle in terms of the processes involved, indicators achieved, and challenges faced. The course participants sounded positive about the conduct of the course but also provided suggestions for improvement.

It is truly a one-of-kind experiment in the history of Indian medical education to introduce a mandatory and completely online research course managed and run by an external agency like the ICMR. It shows the willingness of the medical education regulatory agency to upgrade and adapt to the changing needs of the medical postgraduate education. This is also a step in the direction to improve the quality of research performed by young scientists and their mentors.

Online teaching methods have been shown to be no different than face-to-face teaching methods at the school level. A similar situation is also likely true for adult learning, especially for medical subject matter [23]. Several online teaching courses for nurses, nursing students and practicing doctors have shown good effectiveness when compared to face-to-face teaching [24–28]. A few have shown that online only courses have shown no significant difference [29, 30]. One particular evaluation of an interactive for teaching hypothesis testing concepts was shown to be highly effective [31]. Although these studies point to the fact that online medium of instruction may be beneficial for teaching research methodology to a group of medical personnel, a formal randomized controlled study may be required to prove this conclusively [32].

Similar to our observation dropouts was noticed for several MOOCs organized by university of Virginia [33]. They found a negative relationship between procrastination and achievement in MOOCs. Unlike traditional classroom teaching MOOC enrollment needs fewer requirements for eligibility. The intent for enrollment may reflect a trial and error approach since the cost for initiation of most MOOC’s is small. Thus drop out often tends to be high [34]. In addition, similar to our observation various MOOC’s have reported participant challenges such as digital incompetence and unsupportive environment that could restrain participant’s MOOC experience [35, 36].

Planning for subsequent cycles of the BCBR will incorporate the findings discussed above. Among the many tasks to be undertaken, steps to increase the enrollment coverage, update course contents to reflect recent changes and provide additional relevant reading materials to accommodate the participant feedback are primary. A major step towards developing a college/regional level mentorship plan along the lines of the Medical Education Technology programme of the MCI (now, NMC) is also envisioned. These mentors will be readily available at the college level to clarify learner queries and act as a liaison with the coordination team at ICMR-NIE.

With the experience gained in the first cycle, we were able to introduce certain innovations that provided a richer learning experience to the participants. Participant feedback was instrumental in designing these modifications. Some of the planned modifications for future cycles are lecture specific example accompaniments, Frequently Asked Questions documents, reading materials that are open source and simple enough for a beginner and Live webcast session for real time interaction and query solving. From the administrative perspective, we plan to automate the process of query management by having a chatbot on our website and allowing human interactions for rare and uncommon queries.

In this article, we have provided a framework and a roadmap for anyone who is willing to launch a similar campaign at a national or even a regional level. We hope our experiences will benefit the larger medical education community in designing and delivering such health research MOOCs .

### Limitations

A common enrolment form is used for fetching the details on the SWAYAM portal for all the courses covering engineering, basic sciences, and selected humanities and social sciences subjects. As a result, we could not capture few enrolment details of participants such as the discipline of the medical post graduate studies, institute name, year of enrolment of PG degree. There was paucity of feedback data as we kept it optional for the participants.

## Conclusions

During the inaugural cycle of the online Basic Course in Biomedical Research course, nearly half of the enrolled participants successfully completed and received the certificate. We concluded that India’s MOOC for enhancing capacity in the basics of biomedical research methods among medical PGs and faculty members was a challenging task. The lessons learnt from the inaugural cycle could help in better delivery of the course in the subsequent cycles. Further, the stakeholders need to address larger issues in terms of sustainability and replication in offering similar MOOCs in medical education in India or elsewhere.

### Recommendations

Designing and implementing a MOOC on research methods at the national level is fraught with challenges. Taking into account expected hurdles during the design and planning stage may lead to a seamless experience to the course takers. Technological proficiency of participants with medical background will require intensive training and handholding during the implementation. Experiences from other research methodology related MOOCs need to be reported widely and further studies must be undertaken to enable better implementation and knowledge transfer to the course participants.

## Supplementary Information

**Additional file 1: Figure 1.**Qualitative analysis of feedback received from the participants in a massive open online course on health researchin India, 2019-2020. **Table 1.** Curriculumof Basic Course in Biomedical Research that was aligned with the competencyframework on research methods for medical post-graduates by the NationalMedical Commission. **Table 2. **Profile of participants ina massive open online course on health research in India, 2019-2020 (*n*=24,385). **Table 3. **Participants’ feedback in a massive open online course on health research in India, 2019-2020. **Table 4.**Verbatim examples of technical queries received from the participants in a massive open online course on health research in India,2019-2020

## Data Availability

All data generated or analysed during this study are included in this published article [and its supplementary information files]. The datasets used and/or analysed during the current study available from the corresponding author on reasonable request.
