# Supplementary material for: “Design and implementation challenges of massive open online course on research methods for Indian medical postgraduates and teachers –descriptive analysis of inaugural cycle”
Source: BMC Med Educ. 2022 May 13;22:369. doi: 10.1186/s12909-022-03423-6 (PMC9099314; doi:10.1186/s12909-022-03423-6)
Supplement: Supplementary file 1 — Additional file 1: Figure 1.Qualitative analysis of feedback received from the participants in a massive open online course on health researchin India, 2019-2020. Table 1. Curriculumof Basic Course in Biomedical Research that was aligned with the competencyframework on research methods for medical post-graduates by the NationalMedical Commission. Table 2. Profile of participants ina massive open online course on health research in India, 2019-2020 (n=24,385). Table 3. Participants’ feedback in a massive open online course on health research in India, 2019-2020. Table 4.Verbatim examples of technical queries received from the participants in a massive open online course on health research in India,2019-2020 [file 12909_2022_3423_MOESM1_ESM.docx]

**Additional table 1. Curriculum of Basic Course in Biomedical Research that was aligned with the competency framework on research methods for medical post-graduates by the National Medical Commission**

| **As per NMC format** | | | | | **Basic Course in Biomedical Research** | | | | |
| --- | --- | --- | --- | --- | --- | --- | --- | --- | --- |
| **Number** | **COMPETENCY**  (Student should be able to) | **Domain (K/S/A/C)** | **Level (K/KH/SH/P)** | **Core (Y/N)** | **Session** | **Title** | **Objective of session** | **Teaching/**  **Learning method** | **Assessment method** |
| BRM1.1* | Define research and discuss the underlying values of research. Define spectrum of research from basic (animals, humans, ex and in vitro) applied, translational. Public health | K | K/KH | Y | 1.1 | Introduction to Health research | 1. Describe various dimensions of health research 2. Explain the fundamental principles and key components of  health research process 3. Outline various challenges in designing and implementation of research studies 4. Recognize major issues related to study methods and measurements used in research | Online video, lectures, presentation slides, quizzes and readings for self-directed learning | MCQ |
| BRM1.2 | Describe how to identify and write a research question and discuss hypothesis and objectives | K/S | K/KH/SH | Y | 1.2 | Formulating research question, hypothesis and objectives | 1. Paraphrase a research question 2. Distinguish between descriptive and analytical questions 3. State research hypothesis/es 4. Define research objectives(s) | Online video, lectures, presentation slides, quizzes and readings for self-directed learning | MCQ |
| BRM1.3 | Discuss strategies for literature search | K/S | K/KH/SH | Y | 1.3 | Literature Review | 1. Recognize the importance of performing a literature review 2. Describe the steps in performing a literature review 3. Outline the steps in writing a literature review | Online video, lectures, presentation slides, quizzes and readings for self-directed learning | MCQ |
| BRM2.1 | Describe measures of disease frequency | K | K/KH | Y | 2.1 | Measures of disease frequency | 1. List commonly used measures of disease frequency 2. Define prevalence and incidence 3. Describe uses of prevalence and incidence measures 4. Recognise relationship between incidence and prevalence | Online video, lectures, presentation slides, quizzes and readings for self-directed learning | MCQ |
| BRM 2.2 | Describe descriptive and analytic study designs and discuss their relative merits and demerits | K | K/KH | Y | 2.2 | Descriptive study design | 2.2 Descriptive study design:   1. List the types of descriptive observational study designs 2. Describe the uses of descriptive study designs 3. Recognise key elements of cross sectional study designs | Online video, lectures, presentation slides, quizzes and readings for self-directed learning | MCQ |
| BRM 2.2 | Describe descriptive and analytic study designs and discuss their relative merits and demerits | K | K/KH | Y | 2.3 | Analytical study design | 2.3 Analytical study designs:   1. List the types of analytical observational study designs 2. Recognise key elements of cohort study design 3. Recognise key elements of case-control study design | Online video, lectures, presentation slides, quizzes and readings for self-directed learning | MCQ |
| BRM3.1 | Enumerate and discuss different types of experimental study design and their relative utility | K | K/KH | Y | 3.1 | Experimental study designs: clinical trial | 1. Describe the basic concepts in randomized controlled trials 2. Define the purpose of randomization, blinding and ethical issues related to experimental study design 3. Identity and classify different types of trial designs | Online video, lectures, presentation slides, quizzes and readings for self-directed learning | MCQ |
| BRM3.2* | Describe qualitative research design, tools for data collection discuss how to differntiate them from quantitative designs and the utility and benefits | K | K/KH | Y | 3.3 | Qualitative research design: An overview | 1. Recognize the difference between Qualitative and Quantitative research methods 2. Relate the basic concepts of Qualitative research 3. Describe the methods of data management in Qualitative research | Online video, lectures, presentation slides, quizzes and readings for self-directed learning | MCQ |
| BRM3.3 | Discuss validity of epidemiological studies | K/S | K/KH/SH | Y | 3.2 | Validity of epidemiological studies | 1. Discuss the various errors of measurement in epidemiological studies 2. Distinguish terminologies in epidemiology such as chance, bias and confounding 3. Identify measures to alleviate the errors of measurement in epidemiological studies | Online video, lectures, presentation slides, quizzes and readings for self-directed learning | MCQ |
| BRM4.1 | Describe measurements of study variables | K/S | K/KH/S | Y | 4.1 | Measurement of study variables | 1. List the scales of measurement for different types of data 2. Describe and compute the measures of central tendency 3. Explain the purpose of measures of dispersion 4. Illustrate the advantages and disadvantages of these measures | Online video, lectures, presentation slides, quizzes and readings for self-directed learning | MCQ |
| BRM4.2 | Describe sampling methods and discuss their uses | K | K/KH | Y | 4.2 | Sampling methods | 1. Recognize the importance of sampling in research 2. Distinguish between probability & non probability sampling 3. Discuss the strengths and limitations of each type of sampling method | Online video, lectures, presentation slides, quizzes and readings for self-directed learning | MCQ |
| BRM4.3 | Demonstrate understanding of underlying principles of calculating sample size and power | K/S | K/KH/SH | Y | 4.3 | Calculating sample size and power | 1. Recognize the role of sample size in the power of a statistical test 2. Outline the steps in estimating a sample size 3. Determine the sample size required to estimate population parameters 4. Describe design effect and its influence on statistical power | Online video, lectures, presentation slides, quizzes and readings for self-directed learning | MCQ |
| BRM5.1 | Discuss method of selection of population for various study designs | K | K/KH | Y | 5.1 | Selection of study population | 1. Outline the steps in selecting a study population 2. Define inclusion and exclusion criteria 3. Distinguish between internal and external validity 4. Recognize and address issues related to non-response | Online video, lectures, presentation slides, quizzes and readings for self-directed learning | MCQ |
| BRM5.2 | Describe process of data plan and project management | K | K/KH | Y | 5.2 | Study plan and project management | 1. State the fundamental principles of project management 2. Describe the road map to study planning and management 3. Recognize common reasons for study failures | Online video, lectures, presentation slides, quizzes and readings for self-directed learning | MCQ |
| BRM5.3 | Describe process of designing data collection tools | K/S | K/KH/SH | Y | 5.3 | Designing data collection tools | 1. Explain the various types of data collection tools 2. Describe the components of a data collection tool 3. Define the procedure for construction of a data collection tool 4. Outline the design of a data collection tool | Online video, lectures, presentation slides, quizzes and readings for self-directed learning | MCQ |
| BRM6.1 | Describe principles of data collection | K | K/KH | Y | 6.1 | Principles of data collection | 1. Describe the essential steps in data collection 2. Identify the approaches to ensure data quality during data collection | Online video, lectures, presentation slides, quizzes and readings for self-directed learning | MCQ |
| BRM6.2 | Discuss principles of data management | K | K/KH | Y | 6.2 | Data management | 1. Outline basic structure of a database 2. Identify issues related to data storage 3. Recognize elements of data entry 4. Distinguish types of databases | Online video, lectures, presentation slides, quizzes and readings for self-directed learning | MCQ |
| BRM6.3 | Discuss an overview of data analysis | K/S | K/KH/SH | Y | 6.3 | Overview of data analysis | 1. Describe sequence of data analysis strategy 2. Relate plan of analysis with the nature of research question 3. Outline steps for initial and advanced stages of analysis | Online video, lectures, presentation slides, quizzes and readings for self-directed learning | MCQ |
| BRM7.1 | Describe ethical framework for health research for various study designs | K | K/KH | Y | 7.1, 7.2 | Ethical framework for health research, Conducting clinical trials | Ethical framework for health:   1. Identify the range of ethical issues that need to be addressed in health research 2. Describe the fundamental ethical principles involving human participants 3. List key national and international guidelines and regulations that guide the development and review of research studies 4. Recognize the process and issues related to the conduct of health research and practice of medicine | Online video, lectures, presentation slides, quizzes and readings for self-directed learning | MCQ |
|  |  |  |  |  |  |  | Conducting clinical trials:   1. Recognize the importance of various reviews prior to the implementation of clinical trials 2. Identify critical issues in trial implementation |  |  |
| BRM7.2* | Discuss publication ethics including plagiarism and its consequences | K/S | K/KH/SH | Y | 7.3 | Publication ethics | 1. Recognize various ethical issues related to publication 2. Make use of the guidelines available from various national and international organizations for publication ethics | Online video, lectures, presentation slides, quizzes and readings for self-directed learning | MCQ |
| BRM8.1 | Describe process of preparing a concept paper for research projects | K | K/KH | Y | 8.1 | Preparing a concept paper for research projects | 1. Outline elements of a concept paper 2. Translate research idea into one-page concept paper | Online video, lectures, presentation slides, quizzes and readings for self-directed learning | MCQ |
| BRM8.2 | Describe the elements of a protocol for research studies | K | K/KH | Y | 8.2 | Elements of a protocol for research studies | 1. Describe the various steps in writing a successful research protocol 2. Outline the important components of a research proposal | Online video, lectures, presentation slides, quizzes and readings for self-directed learning | MCQ |

**Additional table 2. Profile of participants in a massive open online course on health research in India, 2019-2020 (n=24,385)**

| **Profile** |  | **Frequency (%)** |
| --- | --- | --- |
| Age (years) | 13-20 | 948 (4.0) |
|  | 21-30 | 15,906 (65.0) |
|  | 31-40 | 5,179 (21.0) |
|  | 41-50 | 1,667 (7.0) |
|  | > 50 | 685 (3.0) |
| Country | India | 24,182 (99.2) |
|  | Ghana | 117 (0.5) |
|  | United States | 11 (0.04) |
|  | Nepal | 8 (0.03) |
|  | Others | 67 (0.3) |
| Educational background | Medical | 16,893 (69.3) |
|  | Engineering | 1,045 (4.3) |
|  | Basic science | 868 (3.6) |
|  | Arts & Humanity | 55 (0.2) |
|  | Commerce & Management | 55 (0.2) |
|  | Others | 5,469 (22) |
| Profession | Post graduate medical student | 13,242 (54.0) |
|  | Faculty | 2,637(11.0) |
|  | Others | 8,506 (35.0) |

**Additional table 3. Participants’ feedback in a massive open online course on health research in India, 2019-2020**

| **Quantitative assessment** | **Mean (SD)** |
| --- | --- |
| **1. Content** |  |
| 1.1 The content was relevant to me (n =1280) | 4.6 (0.6) |
| 1.2 The content was at an appropriate level (n =1291) | 4.5 (0.7) |
| 1.3 The content matched my expectations (n =1295) | 4.4 (0.7) |
| **2. Structure** |  |
| 2.1 The learning objectives were clearly stated (n =1297) | 4.5 (0.7) |
| 2.2 The topic was presented in a logical sequence (n =1293) | 4.5 (0.6) |
| 2.3 The duration of the session was optimal (n =1292) | 4.5 (0.7) |
| **3. Faculty** |  |
| 3.1 The faculty appeared well informed about the subject (n =1294) | 4.6 (0.6) |
| 3.2 The faculty appeared enthusiastic about the subject (n =1297) | 4.5 (0.8) |
| 3.3 The faculty was clear and understandable (n =1289) | 4.5 (0.7) |
| 3.4 The presentation was given at the right pace (n =1291) | 4.5 (0.7) |
| **4. Slides** |  |
| 4.1 The design of the slides was pleasing (n =1290) | 4.5 (0.7) |
| 4.2 The layout of the slides was clear and readable (n =1286) | 4.5 (0.7) |
| 4.3 The graphics used in the slides were appropriate (n =1284) | 4.4 (0.8) |
| **5. General** |  |
| 5.1 This teaching session was of high quality (n = 1292) | 4.5 (0.7) |

**Additional table 4. Verbatim examples of technical queries received from the participants in a massive open online course on health research in India, 2019-2020**

| **Type of queries** | **Solutions planned and implemented** |
| --- | --- |
| ***Enrolment related***  “I had completed my post-graduation in nursing. Can I do this online basic course on biomedical research?”  “I am unable to trace my user id and password”  “I have been trying to submit the online enrolment form but it’s not getting accepted” | - Basic details of the course, eligibility, course process, timelines, FAQs, and contact information added in the website - Tutorials video developed for enrollment, forget username and password and assignment submission and shared via course page - Clarifications sought from course coordination committee on eligibility of some qualifications - Working phone line to answer queries established (on working days from 9 AM to 5:30 PM) - Participant relations executive appointed for handling large volume of email queries |
| ***Exam related***  “From where I can get study materials for examination preparation?”  “I am finding it difficult to pay for the exam registration”  “I have wrongly entered exam date”  “How do we know that our registration is accepted?”  “I'm unable to select city in exam centre”  “Is there a minimum marks limit for passing the exam?”  “Spelling of my name is written wrong and photo upload is not proper- how to rectify?” | - Tutorials video developed for enrollment, forget username and password and assignment submission and shared via course page - Working phone line to answer queries established (on working days from 9 AM to 5:30 PM) - Participant relations executive appointed for handling large volume of email queries - Queries requiring NPTEL support forwarded and resolved |
| ***Course related***  “I was unable to access the lectures and assignments”  “How can I Access the discussion forum?”  “How to get the assignments online”  “I have not received any email regarding my assignment score  “I’m not able to open assignments”  “Due to heavy schedule in residency I couldn't complete my assignments- can you please extend the date?”  “Quiz assignments are not getting submitted” | - Tutorials video developed for assignment submission and discussion forum shared via course page - Working phone line to answer queries established (on working days from 9 AM to 5:30 PM) - Participant relations executive appointed for handling large volume of email queries |

**Additional figure 1. Qualitative analysis of feedback received from the participants in a massive open online course on health research in India, 2019-2020**
